# ActivePPI: quantifying protein–protein interaction network activity with Markov random fields

**DOI:** 10.1093/bioinformatics/btad567

**Published:** 2023-09-12

**Authors:** Chuanyuan Wang, Shiyu Xu, Duanchen Sun, Zhi-Ping Liu

**Affiliations:** Department of Biomedical Engineering, School of Control Science and Engineering, Shandong University, Jinan, Shandong 250061, China; Department of Biomedical Engineering, School of Control Science and Engineering, Shandong University, Jinan, Shandong 250061, China; School of Mathematics, Shandong University, Jinan, Shandong 250100, China; Department of Biomedical Engineering, School of Control Science and Engineering, Shandong University, Jinan, Shandong 250061, China

## Abstract

**Motivation:**

Protein–protein interactions (PPI) are crucial components of the biomolecular networks that enable cells to function. Biological experiments have identified a large number of PPI, and these interactions are stored in knowledge bases. However, these interactions are often restricted to specific cellular environments and conditions. Network activity can be characterized as the extent of agreement between a PPI network (PPIN) and a distinct cellular environment measured by protein mass spectrometry, and it can also be quantified as a statistical significance score. Without knowing the activity of these PPI in the cellular environments or specific phenotypes, it is impossible to reveal how these PPI perform and affect cellular functioning.

**Results:**

To calculate the activity of PPIN in different cellular conditions, we proposed a PPIN activity evaluation framework named ActivePPI to measure the consistency between network architecture and protein measurement data. ActivePPI estimates the probability density of protein mass spectrometry abundance and models PPIN using a Markov-random-field-based method. Furthermore, empirical *P*-value is derived based on a nonparametric permutation test to quantify the likelihood significance of the match between PPIN structure and protein abundance data. Extensive numerical experiments demonstrate the superior performance of ActivePPI and result in network activity evaluation, pathway activity assessment, and optimal network architecture tuning tasks. To summarize it succinctly, ActivePPI is a versatile tool for evaluating PPI network that can uncover the functional significance of protein interactions in crucial cellular biological processes and offer further insights into physiological phenomena.

**Availability and implementation:**

All source code and data are freely available at https://github.com/zpliulab/ActivePPI.

## 1 Introduction

Protein–protein interactions (PPI) are among the most crucial molecular cooperation events in cells and important targets for therapeutic intervention in disease (Zhou *et al.* 2022). A comprehensive understanding of the mechanism of PPI will help to interconnect the diverse proteins in complex pathways to understand the signaling rules in cell systems (Gao *et al.* 2023). Currently, some high-throughput techniques, including yeast two-hybrid technology (Gavin *et al.* 2002), tandem-affinity purification (Gavin *et al.* 2002), high-throughput mass spectrometry protein complex identification (Ho *et al.* 2002), and Co-Immunoprecipitation (Johnson *et al.* 2021), have significantly contributed to the generation and investigation of PPI across various species (Lee *et al.* 2018). However, the acquisition of PPI is impeded by the constraints of experimental environment and equipment resolution, resulting in a time-consuming and laborious process and an elevated rate of false positives (Mrowka *et al.* 2001, Lenz *et al.* 2021). Numerous data-driven computational methods have emerged in recent years, enabling the rapid characterization of unknown PPI (You *et al.* 2017, Gligorijević *et al.* 2021). The majority of these bioinformatics methods rely on knowledge-based interactions as benchmarks for performance validation. Although the available PPI have emerged as significant resources for investigating and providing insights into protein complex functions, most of them contain no specific cellular environment information. Thus, the actual roles and activities of these interactions remain uncertain in diverse phenotypic conditions. For any recorded PPI, it is crucial to determine their activity in different cellular contexts and stimuli.

As an unbiased and global quantitative method, mass spectrometry (MS)-based proteomics offers excellent opportunities for comprehensive analysis of PPI. Complex proteomics data extracted by MS quantify protein abundance information and characterize the internal environmental state of cells (Cravatt *et al.* 2007, Aebersold and Mann 2016). Moreover, MS is capable of interpreting the intercellular specific state of protein complexes.

However, the activities of PPI on different MS data are unclear although they could detect their interactions. This active state explains their ability to generalize cellular microenvironments. For example, Chen *et al.* (2019) revealed the key pathways of pan-cancer subtypes based on protein MS data; Selevsek *et al.* (2015) analyzed SWATH-MS data enrichment and quantified significantly regulated metabolic pathways in *Saccharomyces cerevisiae*; Gao *et al.* (2023) introduced a double-viewed hierarchical graph learning model HIGH-PPI to predict PPI and details. It is still insufficient and inadequate to provide insight into the PPIN activity in the specific cellular environment. Even with the PPI documented in different knowledge-bases, they contain no information about specific cellular environments. The binary interaction event only indicates how they “can” be interacted with each other and the condition-specific role or activity of these interactions remains uncertain. Therefore, in the pathway or network analysis, an important prerequisite is to understand the activity of PPIN given a specific cellular condition. This activity suggests that proteins may elicit different functions in response to different cellular environments. Exploiting prior knowledge of protein interaction networks is expected to reveal the active PPIN under specific conditions and phenotypes.

So far, gene set enrichment analysis can be implemented to delineate the activity of a given gene set at a specific cellular condition. These user-defined or preset gene sets reflect the functional differences in different phenotypes. Generally, the available gene set enrichment analysis methods can be categorized into two groups. First is the set-based models that ignore network structure: CAMERA (Wu and Smyth 2012), GSA (Efron and Tibshirani 2007), GSEA (Subramanian *et al.* 2005), GSVA (Hänzelmann *et al.* 2013), ORA (Goeman and Bühlmann 2007), and SAFE (Barry *et al.* 2005). Second is the network-based models that incorporate networking interactions between genes: GANPA (Fang *et al.* 2012), GGEA (Geistlinger *et al.* 2011), LPIA (Pham *et al.* 2011), NEAT (Signorelli *et al.* 2016), PathNet (Dutta *et al.* 2012), and SPIA (Tarca *et al.* 2009). In contrast, network activity evaluation is a more specific interpretation of the activity of multiple genes.

Specifically, several studies have begun to quantify the activity of a given network in some specific cellular environments characterized by gene expression profiles. For instance, Liu *et al.* (2013) calculated the probability of concordance between a reference gene network and a gene expression profile by the network architecture screening (NAS) method. NAS has achieved satisfactory results in mouse circadian regulation data. Wang *et al.* (2022) established a network activity evaluation (NAE) framework based on the dynamic Bayesian network, and it comprehensively evaluated the consistency between gene regulatory networks and gene time-course expression profiles. In addition, some studies have verified the relationship between molecular regulation and the environments with biological experiments (Gutierrez-Rios *et al.* 2003, Larsen *et al.* 2019). However, they are not scalable and difficult to be extended for PPIN. To our best knowledge, the evaluation of network activity at the protein level has not yet been available. Therefore, it is urgent to propose a framework to quantify the network activity evaluation for PPIN in specific tissues and contexts.

In this article, we propose a computational framework called ActivePPI for quantifying the activity of a given PPIN in protein MS abundance data. ActivePPI models PPIN as Markov random field (MRF) by assuming that the state transition process of a protein is only affected by the interaction of its neighboring proteins in the network. The energy of each fully connected subgraph (also called maximal clique) within the network is estimated based on the conditional independence of proteins. The energy function reflects the closeness of the probability density distribution within each cluster in some particular cellular environment and phenotypic condition, which reflected by the interactions between proteins. Furthermore, we evaluate the degree of agreement between PPIN structure and MS abundance data using a nonparametric testing procedure for deriving an empirical *P*-value.

To show the assessment of PPIN activity in specific condition, we perform numerical experiments on simulation data, breast cancer data, and SARS-CoV-2 data to validate the efficiency and versatility of ActivePPI. Compared with the other alternative strategies and state-of-the-art algorithms, ActivePPI performs satisfactorily competitive results. Based on MS data, ActivePPI not only quantifies the activity value of a given network, but also searches for the optimal network architecture to match the current condition reflected by the measurement data. In addition, the estimation of the pathway activity by ActivePPI is also similar to the significant degree of the pathway enrichment. Comparing with other protein ensembles, it indicates whether the biological processes triggered by this pathway are significant in some specific cellular environment and phenotypic condition.

## 2 Materials and methods

### 2.1 ActivePPI framework

**Figure 1.**
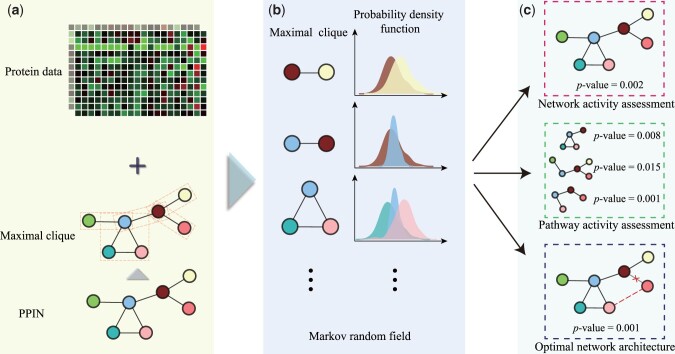
Overview of ActivePPI. (a) Processes protein mass spectrometry data and factorizes PPIN into clusters according to Markov properties. (b) Computes the energy of each maximal clique by estimating the density distribution of proteins. (c) Rewires the network multiple times to calculate the empirical *P*-value for the evaluating PPIN, in the form of network activity, pathway activity, and optimal network architecture.

In this work, we propose an MRF-based method, ActivePPI, to quantify the activity of a prior PPIN with proteomics measurement data. In specific phenotypic condition and cellular microenvironment, proteins will interact each other for performing specific functions in form of a PPIN. We formulate a protein with its neighboring ones with an MRF model. The state transition is due to the information transfer of the existence of interacting proteins under the one-step Markov property assumption. This leads to the similarity of their mass spectrum abundance in the data density distribution. Figure 1 illustrates the workflow of the ActivePPI model. First, the PPIN is decomposed into multiple maximal cliques according to the MRF. Then, the energy of each maximal clique is estimated independently to further obtain the joint probability density of the global network. Finally, an empirical *P*-value is derived by multiple permutation tests to quantify the statistical significance of the agreement between the evaluating network architecture and the MS profiling data.

### 2.2 Data

To verify the performance of ActivePPI, three types of datasets, i.e. simulation datasets, breast cancer (BRCA), and SARS-CoV-2 dataset, are used in the experiments.

#### 2.2.1 Simulation datasets

**Figure 2.**
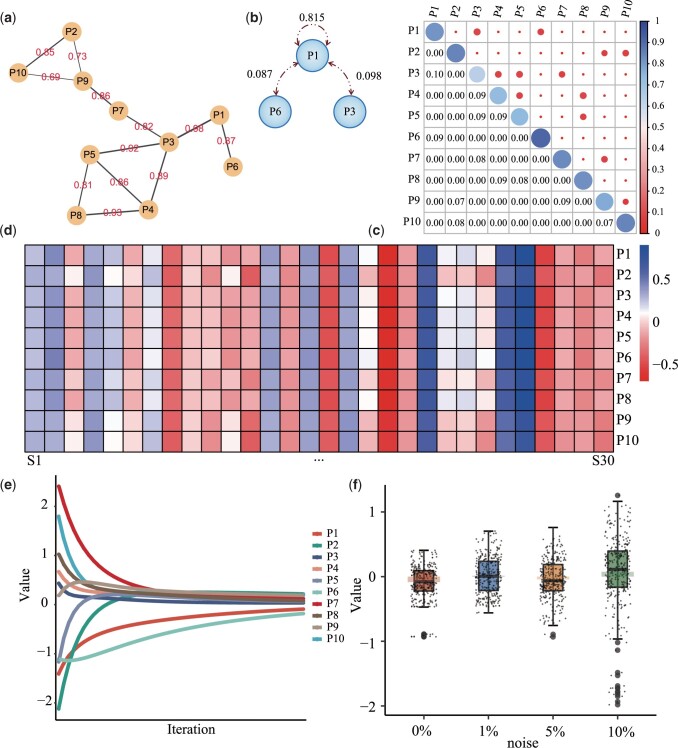
Details of a toy dataset. (a) Simulation example of PPIN, (b) state transition process related to protein P1, (c) state transition probability matrix of all proteins, (d) convergence of protein simulation data, (e) expression values of simulated proteins in steady state, and (f) data distribution under different noise pollution strength.

PPIN arises from spontaneous information transitions between proteins in some specific cellular environment, enabling cell diversification and promoting heterogeneity progression. According to the Markov properties, PPIN is constructed as shown in Fig. 2a, and the strength of the interaction relationship will be refined further. Then, based on the edge weights, we simulate the state transition course that occurs between 10 proteins, and the sum of the state transition probabilities of each protein is 1. The state transition probability of protein P1 is defined as shown in Fig. 1b. Moreover, Fig. 2c is the state transition matrix of all proteins, and we verify the Markovian state transition process to be convergent by calculating the matrix eigenvalues. The protein mass spectrum at time *t*0 is randomly initialized with under the Gaussian distribution $\sigma \left(0,\mu_{0}\right)$ with mean 0 and variance $\mu_{0}=1$, and is further updated according to the following equation until convergence.

(1) $$\;I^{t+1}\left(i\right)=I^{t}\left(i\right)\times \theta \left(i,i\right)+\sum_{j\in N}I^{t}\left(j\right)\times \theta \left(i,j\right)+\sigma \left(0,\mu \right),$$

where *N* represents the neighbor set of the *i*th protein, θ and σ are the state transition probability matrix and Gaussian noise with variance μ, respectively.

Figure 2d depicts the iterative process of 10 proteins based on Equation 1, and the MS abundance of protein profiles eventually reaches a plateau as time progresses. Updates are stopped at iteration 50, and the stable protein state is used for further analysis. This biological process is randomly repeated 30 times to obtain more available samples, and finally the MS abundance data of 10 proteins and 30 samples is obtained as shown in Fig. 2d. Furthermore, according to the variance $\mu =\left\{1\%\mu_{0},\;5\%\mu_{0},\;10\%\mu_{0}\right\}$, we further simulate protein MS data “Simu1”, “Simu2”, “Simu3”, and “Simu4” contaminated by different scales of Gaussian noise. The distribution of these data is shown in Fig. 2f. As the degree of noise-pollution deepens, the fluctuation range of the data becomes larger, and more outliers emerge.

#### 2.2.2 BRCA and SARS-CoV-2 datasets

Further, we analyzed the activity of the PPIN based on the real MS datasets of BRCA and SARS-CoV-2 samples. In detail, we downloaded and preprocessed protein MS abundance data from 133 breast tumor samples and adjacent normal tissues (including 18 participants) of 125 breast cancer patients. The MS profiling information of 10 491 proteins in 151 samples was retained after the data were completed by K-Nearest Neighbors and normalized. A summary report of these samples and proteins can be found at PDC (https://proteomic.datacommons.cancer.gov/pdc/) database with accession number PDC000120 (Mertins *et al.* 2016, Krug *et al.* 2020). Moreover, we extracted protein MS data from 144 autopsy reports of 7 organs from 19 COVID-19 patients. The MS data quantifies 11 394 proteins from 144 patients and 74 controls (Nie *et al.* 2021). After preprocessing, the MS information of 143 patients and 73 normal subjects on 7087 proteins was retained.

To analyze the activity of pathways, we downloaded a total of 2624 pathways from the MSigDB database (including the Reactome library and WikiPathway library) and the KEGG database (https://www.genome.jp/kegg/) (Subramanian *et al.* 2005). Furthermore, each pathway is constructed with PPIN based on protein interactions deposited in String and InWeb_IM databases (Li *et al.* 2017, Szklarczyk *et al.* 2023). In addition, redundant and isolated nodes in the network and pathway were removed based on the quantified protein profiles in BRCA and SARS-CoV-2 data, respectively, and unmeasured protein nodes were also removed. Besides, some networks and pathways were ignored if they have less than 2 or more than 200 proteins. Finally, the BRCA dataset obtains 2385 pathways and networks, in which the number of nodes ranges from 2 to 188 and the number of edges varies from 2 to 23 161; the SARS-CoV-2 dataset obtains 2261 pathways and networks, in which the number of nodes ranges from 2 to 188, and the number of sides is from 2 to 39 448 (Table 1).

**Table 1.** Pathways related to BRCA and SARS-CoV-2.

	Pathway	Synopsis	Database	No. of Protein
BRCA	M27888	Estrogen-dependent gene expression	Reactome	87
	M39739	Breast cancer	WikiPathways	91
	hsa05224	Breast cancer	KEGG	81
SARS-CoV-2	M39859	Type I interferon induction and signaling	WikiPathways	18
	M40066	Mitochondrial immune response	WikiPathways	20
	M41727	Genome sequence of SARS-related coronavirus	Reactome	50
	M42560	Antiviral and anti-inflammatory effects	WikiPathways	15
	M42569	SARS-CoV-2 signaling pathway	WikiPathways	117
	M42580	T-cell activation	WikiPathways	40
	M42581	The antagonistic effects of the SARS-CoV-2 variant on innate immune activation	WikiPathways	6
	hsa05171	Coronavirus disease	KEGG	159

### 2.3 Markov random fields

Define G=(V,E) as a PPIN with*n* nodes, $X=\left\{x_{1},x_{2},\ldots ,x_{n}\right\}$ as the corresponding MS abundance information defined on V, and each node contains *s* samples. This graph can be modeled as a Markov network under the following conditions. The joint distribution function P(x) consisting of a set of potentials ϕ is derived.

- Positivity: P(x)>0,∀x∈X,
- Markovianity: $P(x_{i}|x_{\left\{-i\right\}})=P(x_{i}|x_{N\left(i\right)})$,

where X is also interpreted as the collection of potential states of a series of proteins.

In a Markov network, the state of $x_{i}$ is determined only by its associated protein set $x_{N\left(i\right)}$, where N(i) is all neighbors of vertex *i* in graphG. The potential function ϕ(⋅) assigns the value to each maximally connected clique in the MRF. According to the Hammersley-Clifford theorem, the following probability density decomposition can be obtained

(2) $$P(x)=\frac{1}{Z}\prod_{c\in C}\phi \left(x_{c}\right),$$

where *C* represents the set of maximal cliques in the graph. Since $\phi \left(x_{c}\right)$ does not need to be used as the probability value when representing a potential function, *Z* can be used as the normalized score to control the joint distribution probability of the entire state to 1. To ensure its nonnegativity and controllable precision, we introduce the energy function E(x) to define the probability distribution of the output variable *x*.

(3) $$P(x)=\frac{1}{Z}\text{exp}\;\left(-E\left(x\right)\right),$$

(4) $$E\left(x\right)=\sum \phi \left(x_{c}\right).$$

From the above equations, we can reason about the conditional independence relationship between random variables. Specifically, we can conclude that different maximal cliques are independent, while random variables within the same clique are highly likely to be dependent on each other.

### 2.4 Energy estimation

The distributions of MS profiles for protein sets with distinct functions exhibit significant differences. Due to prolonged interactions within the cellular environment and condition, proteins with strong association properties will exhibit a slight degree of consistency in their data distribution. Moreover, the activity of this interaction relationship in the specific condition can be further calculated after being amplified by Markov network and power function. Specifically, ActivePPI firstly estimates the density of each protein according to the Gaussian kernel density estimator (kde), which is defined as follows:

(5) $$\mathrm{kde}\left(x_{p}\right)=\frac{1}{sh}\sum_{i=1}^{s}\frac{1}{\sqrt{2\pi }}e^{-\frac{1}{2}{\left(\frac{x_{p}-x_{p,i}}{h}\right)}^{2}},$$

where $x_{p}$ contains *s* sample information of protein *p*. Furthermore, the parameter *h* is the kernel bandwidth that can be estimated by the scheme of Sheather *et al* (Sheather and Jones 1991). After determining the bandwidth, the density of each point can be estimated approximately.

The protein distribution $\mathrm{kde}\left(x_{1}\right),\ldots ,\mathrm{kde}\left(x_{n}\right)$ in the biological condition can be estimated by Equation 5. Moreover, PPI are stored as networks and reflected inside clique. There is mutual influence between proteins of the same clique, which leads to local similarities between their data densities. Furthermore, the distance between proteins inside the clique is estimated by an optimized measurement function, i.e.

(6) $$\phi \left(x_{c}\right)=\sum_{i,j\in c,i\neq j}\ln\left(1+D\left(x_{c(i)},x_{c(j)}\right)\right){\left(n_{c}{}^{2}-n_{c}\right)}^{-1},$$

(7) $$D\left(x,y\right)=\mathrm{dist}\left(\mathrm{kde}\left(x\right),\mathrm{kde}\left(y\right)\right)\cdot l^{-1},$$

where *c* is a clique containing $n_{c}$ proteins, and *l* is the total amount of clique to balance the network of different connections. And dist(⋅) is defined as Euclidean distance, which can be modified according to the characteristics of the data. In addition, we also recommend using four additional distance measurement tools including Cosine, Canberra, Manhattan, and Minkowski. Equation 7 estimates the distance between pairs of protein density distributions when the number of proteins inside the clique exceeds 1, otherwise the potential function is set to 1. If the interior of the clique is unstable, i.e. the network structure is not suitable for this specific condition, then the potential function $\phi \left(x_{c}\right)$ will be very large.

### 2.5 Evaluation criteria and parameters

By estimating all protein states and the normalization factor *Z*, the joint probability density of the Markov network can then be calculated, but this process is usually difficult to achieve perfectly. Furthermore, the goal of the ActivePPI model is not to acquire a precise joint probability density, but rather to assess the PPIN activity in specific cellular environments and phenotypic conditions by calculating the relative probability density. Therefore, we set the normalization factor *Z* to 1 for the convenience of calculation, and numerically quantify the statistical significance of PPIN by randomly rewiring the network linages multiple times. The detailed refactoring rules are determined by the network rewiring function in the R package igraph (Maslov and Sneppen 2002, Milo *et al.* 2003). The actual activity of each PPIN in this condition is calculated by ActivePPI. Ideally, the joint probability density value of the evaluating network is significantly different from that of the random network. When the optimal network architecture is the evaluating network, its joint probability density is expected to be the lowest one according to Eq. 2. Based on nonparametric testing procedure, we derive an empirical *P*-value to quantify the statistical significance of networks by the following equation.

(8) $$\begin{array}{c} P-\mathrm{value}=\frac{1}{\tau }\sum_{i=1}^{\tau }I\left(P\left(Net_{i}\right),P\left(Net_{\mathrm{true}}\right)\right),\\ \text{where}\;\;I(x,y)=\{\begin{array}{c} 1,\;\;\;if\;x<y,\\ 0,\;\;\mathrm{otherwise}, \end{array} \end{array}$$

where τ is the number of times the network has been rewired, and it is set to 1000 in our experiments. $P\left(Net_{\mathrm{true}}\right)$ and $P\left(Net_{i}\right)$ are the joint distribution probability values of the original evaluating network and the random network respectively. We distinguish the significance of the network with a threshold of 0.05, the smaller the *P*-value, the more significant the role of PPIN in the MS profile of the protein.

## 3 Results and discussion

This section presents the results and discussion of implementing the proposed ActivePPI method on the simulation datasets, BRCA and SARS-CoV-2 datasets, respectively.

### 3.1 Network activity assessment

The results on simulation data are listed in Table 2. In the simulation study, the PPIN is rewired 1000 times on these 4 simulated datasets. Furthermore, the joint probability density under different distance metrics is calculated by ActivePPI and an empirical *P*-value is derived to quantify the significance of the gold standard network with the simulation data.

**Table 2.** Evaluation results of ActivePPI on simulation data.

Run	ActivePPI (*P*-value±standard deviation)				
	Canberra	Cosine	Euclidean	Manhattan	Minkowski
Simu1 (0% noise)	0.007±0.002	0.008±0.002	0.007±0.002	0.007±0.002	0.007±0.003
Simu2 (1% noise)	0.007±0.002	0.007±0.002	0.008±0.003	0.008±0.002	0.008±0.003
Simu3 (5% noise)	0.008±0.002	0.008±0.003	0.008±0.002	0.012±0.003	0.008±0.002
Simu4 (10% noise)	0.007±0.002	0.007±0.003	0.011±0.002	0.011±0.003	0.011±0.003
Mean	0.007	0.008	0.009	0.010	0.009

In principle, the gold standard network is expected be the most significant network, i.e. *P*-value = 0.001. However, during the operations of the algorithm, technical noise can affect the accuracy of activity assessment for the evaluating network, causing it to fluctuate within an acceptable range of accuracy. The ActivePPI method can more accurately assess the actual role of PPIN in protein MS profiling data. Furthermore, under 1%, 5%, and 10% noise pollution, the task of network activity assessment becomes more difficult due to the deterioration of data quality. As the noise pollution becomes stronger, the accuracy of ActivePPI for evaluating the reference network decreases, but still maintains satisfactory results (*P*-value = 0.011) even under 10% noise pollution.

Based on different settings, the proposed ActivePPI method can quantify the activity of PPIN under different conditions. By rewiring the network multiple times, ActivePPI is able to remove false-positive interactions in the prior PPIN, and retaining the PPIN structures for which the response relationship occurs in the protein profiling condition. The optimal network architecture and its numerical activity values can provide auxiliary biological insights for revealing the function of protein interactions in a specific condition.

### 3.2 Pathway activity assessment

So far, there are few applicable algorithms to evaluate the activity of PPIN in different MS datasets, and it is difficult to compare the performance of ActivePPI with theirs directly. However, interaction relationships activated in the condition-specific environment are often due to functional activation of proteins. Moreover, the proteins linked by these interactions are more characteristic of the data than other proteins. That is, protein sets in the active network are often important compared to other protein sets. Therefore, the ActivePPI model can be used for pathway activity analysis based on network activity assessment results.

Moreover, 12 protein set activity analysis methods are performed for comparative study, which can be divided into two types: set-based models, i.e. CAMERA, GSA, GSEA, GSVA, ORA, and SAFE; network-based models, i.e. GANPA, GGEA, LPIA, NEAT, PathNet, and SPIA. The details of these methods are listed in Supplementary Table S1.

**Figure 3.**
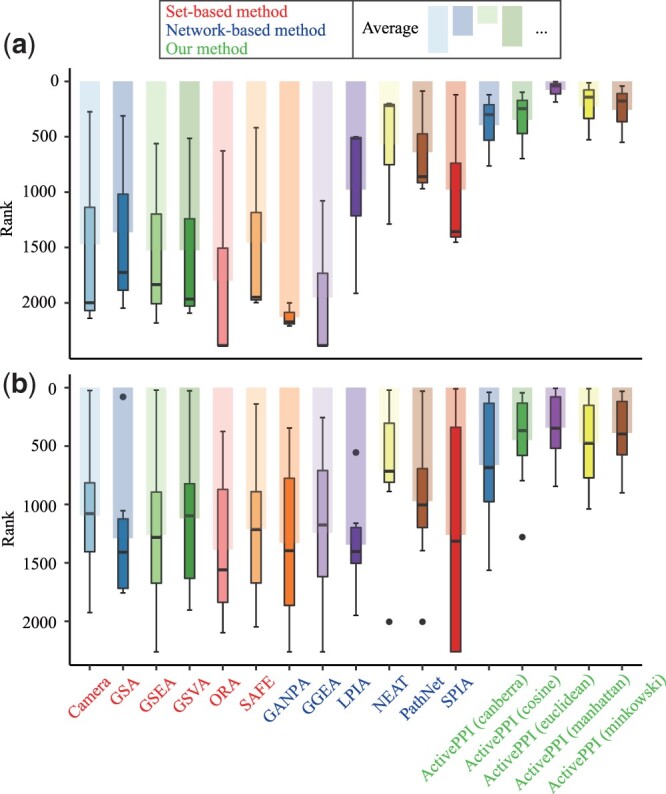
Pathway activity assessment results of all comparing methods, “Rank” refers to the ranking of disease-related pathways in (a) BRCA, (b) SARS-CoV-2.

All methods perform pathway activity assessment on BRCA and SARS-CoV-2 datasets, respectively. These algorithms are run with default parameters and permuted 1000 times if requested. Notably, PPIN is undirected and all *P*-values are corrected by Benjamini–Hochberg method. Further, pathways are sorted according to their *P*-values, and ActivePPI uses distance measures to refine the detailed order for pathways with the same *P*-value (the closer the distance, the smaller the order). Figure 3 shows the statistical results related to BRCA or SARS-CoV-2 datasets, and the smaller the Rank value, the better the performance of the algorithm. Also, the complete ranking results are listed in Supplementary Table S2. Our summary for these results are as follows:

1. The ActivePPI model outperforms other methods notably because it evaluates the specific network for the information transition between proteins. The average Rank value of the ActivePPI model is 378 under different parameters, which is significantly better than other models (from 636 to 1728), and it reaches the optimum when the distance parameter is “Euclidean”. Furthermore, the rules of interaction need to be tailored to the particular biological condition, and only ActivePPI of these comparing models takes this into account. The remaining methods are more sensitive to specific pathways and have obtained satisfactory results. But on average, they do not achieve the impressive results because they can only be applied to their application scenario.
2. The pathway activity analysis of ActivePPI verifies the robustness of the proposed model under five different measurement metrics. In the BRCA dataset, the average rank of the model ranges from 76 to 394 with a standard deviation of 83.23; in the SARS-CoV-2 dataset, the average rank of the model ranges from 388 to 662 with a standard deviation of 91.56. For both datasets, the model can accurately rank the relevant pathways, and the performance fluctuation under different parameters is also acceptable.
3. Generally speaking, the network-based model of network evaluation is better than the set-based model due to the reasonable import of network information. The average rank value of the network-based model is 1134, which is better than the average rank value of 1381 for the set-based method. Furthermore, the best method is NEAT, which has mean results of 568 and 703 in BRCA and SARS-CoV-2 datasets, respectively. The NEAT method introduces the number of links in PPIN to verify whether there is a hypergeometric distribution of the enrichment results, but there are still some shortcomings due to its failure to make full use of the MS abundance data.

In summary, these existing methods achieve the network activity assessment, but they are still insufficient. Moreover, they cannot fully adapt to the pathway activity analysis of proteomic data. The results prove that ActivePPI has satisfactory performance in assessing PPIN activity and is applicable to protein MS data in flexible and different experimental settings.

### 3.3 Optimal network architecture

To verify the performance of ActivePPI in the real cellular condition, it is used to reveal the activated PPI in the M42560 pathway during SARS-CoV-2 virus infection. We run the ActivePPI algorithm on this pathway and return the activity values of the evaluating network and the optimal PPIN architecture based on the SARS-CoV-2 MS data.

**Figure 4.**
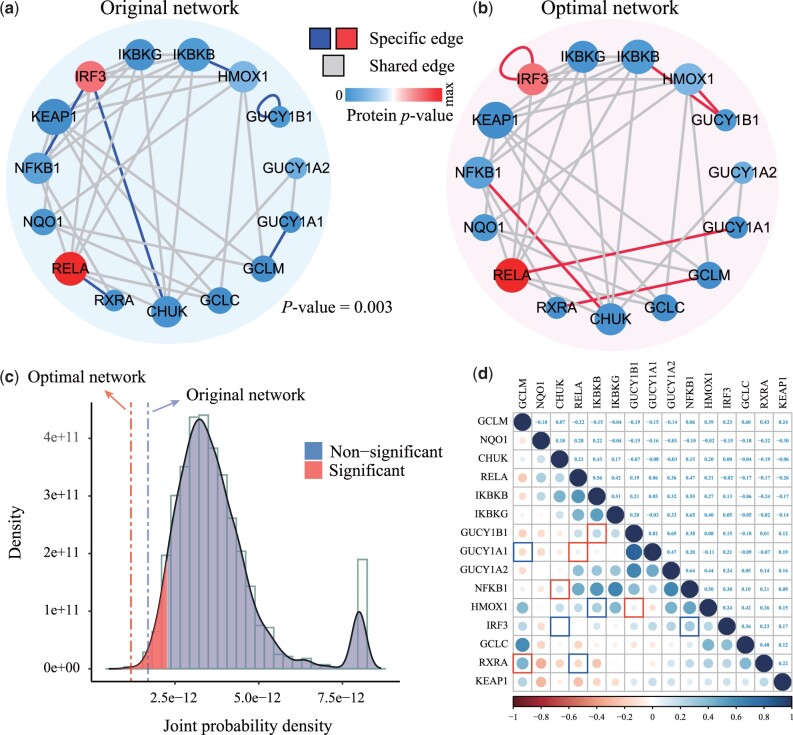
Results of ActivePPI evaluation on Pathway M42560. (a) The evaluating network of M42560, the *P*-value assessed by ActivePPI is 0.003. The network is significant but not optimal. (b) The optimal network architecture of M42560 has six edges rewired compared with the original network, and these edges are significant matching protein MS data for SARS-CoV-2. (c) Joint probability density of all random networks, with significant networks marked. (d) Pearson correlation coefficients are calculated based on the MS data, and the specific edges of the evaluating network and the optimal network are marked.

The details of the activity evaluation results are shown in Fig. 4a–c, and the *P*-value of the evaluating network is 0.003. Figure 4a and b are the original PPIN recorded in the knowledgebase and the optimal PPIN architecture corrected by ActivePPI (with the lowest joint probability density in permutation), respectively. We color-code the *t*-test results for normal and case states for all proteins. Furthermore, Fig. 4c shows the statistical histograms and density curves of all random networks, and distinguishes the significance depending on the threshold. The *P*-value of the evaluating network is lower than the threshold of 0.05, which proves that the dysfunction of M42560 pathway is closely related to SARS-CoV-2 infection (Dömling and Gao 2020, Gao *et al.* 2020).

Specifically, M42560 contains 90 interactions between 15 proteins. These interaction edges are all derived from the Covid-unspecified public knowledgebase, and some of these prior protein interactions may not be active during the SARS-CoV-2 infection. A total of 6 pairs of interaction relationships are revealed and corrected by ActivePPI, covering a total of 10 proteins including CHUK, GCLM, GUCY1A1, GUCY1B1, HMOX1, IKBKB, IRF3, NFKB1, RELA, and RXRA. Furthermore, the pairwise relationship between the corresponding proteins is calculated by Pearson correlation coefficient (PCC). And, the specific relationship between the evaluating original PPIN and the optimal network is marked blue (or red) as shown in Fig. 4d. These correlations can intuitively reflect the kinetic responses of specific PPIN between proteins during SARS-CoV-2 infection.

Taking the protein RXRA as an example, RXRA interacts with RELA proteins in the compiled evaluating network, and the correlation between the two proteins is –0.17; while in the optimal network architecture, RXRA interacts with GCLM, and the correlation between these two proteins is 0.43. The correlation of the latter is significantly higher than that of the former, suggesting that the RXRA-GCLM interaction revealed by ActivePPI is more adaptable to the current cellular environment of viral infection. Furthermore, the *t*-test *P*-value for GCLM and RELA in this specific cellular setting are 4e–5 and 0.79, respectively. This further verifies that GCLM may interact more likely than RELA during this viral infection.

Overall, the protein set activity analysis results obtained by ActivePPI can reflect the interactions and biological mechanisms of different pathways within condition-specific proteomic data. Moreover, the specific PPIN validated by ActivePPI can provide deeper insights into the kinetic response during SARS-CoV-2 viral infection, and are also expected to provide useful suggestions for studying PPIN in diverse phenotypic conditions and cellular environments.

## 4 Conclusions

In this article, we proposed ActivePPI, a flexible framework for quantify the activity of a prior PPIN under certain condition with MS profiling data. The PPI deposited in the knowledgebases contain no information about specific cellular environment and phenotypic condition. It is unclear how they perform their orchestrated functions in different conditions. For a condition-specific MS profiler, ActivePPI can realize the tasks of network activity evaluation, pathway activity assessment and optimal network architecture identification. First, ActivePPI models the state transition process among proteins as a Markov random field. Then, the PPIN is decomposed by Markov network property and multiple maximal cliques are obtained. The interior of the clique interacts with each other and has the consistency of density distribution, while the maximal cliques have conditional independence between each other. Finally, the joint probability density of the global PPIN is estimated based on the internal energy of each maximal clique. Furthermore, by randomly initializing the network multiple times, ActivePPI is able to compare the probability density value of the responses with that of the evaluating network to quantify an empirical *P*-value of statistical significance.

Numerical experiments on simulated examples, real diseases such as BRCA, and SARS-CoV-2 demonstrate the proposed pipeline is effective and efficient in quantifying the PPIN activity from protein MS profiling data. Meanwhile, ActivePPI revealed the protein interaction mechanism during BRCA progression and SARS-CoV-2 virus infection. In short, ActivePPI is a scalable proteomic data analysis framework that integrates network activity evaluation, pathway activity assessment, and optimal network architecture tuning, which is expected to provide some feasible guidance for analyzing PPIN and protein MS data. In the future, we will continue to improve ActivePPI for assessing PPIN activity. Moreover, we envision incorporating information like protein structure and sequence to facilitate the implementation of multi-view network dynamics modeling for PPIN. This endeavor is expected to reveal the mechanisms behind protein state transitions with more comprehensive information.

## Supplementary Material

btad567_Supplementary_Data

## Data Availability

The simulation data used in this article are available at GitHub (https://github.com/zpliulab/ActivePPI). The protein mass spectrometry source files for BRCA are available in PDC database with accession number PDC000120. The source data for COVID-19 patients are available in the supplementary files of the original paper (https://doi.org/10.1016/j.cell.2021.01.004)
