# Supplementary material for: ActivePPI: quantifying protein–protein interaction network activity with Markov random fields
Source: Bioinformatics. 2023 Sep 12;39(9):btad567. doi: 10.1093/bioinformatics/btad567 (PMC10516639; doi:10.1093/bioinformatics/btad567)
Supplement: btad567_Supplementary_Data [file btad567_supplementary_data.pdf]

Supplementary Information for

ActivePPI: Quantifying Protein-Protein Interaction Network Activity with Markov Random Fields

Chuanyuan Wang, Shiyu Xu, Duanchen Sun and Zhi-Ping Liu

Table S1. Details of the comparison methods.

Method	Abbreviation	Description	Topology required?
Correlation adjusted mean rank gene set test	Camera	Characterize gene set ordering based on correlation between genes by estimating variance inflation factor, avoiding false positive adjustments.	No
Gene Set Analysis	GSA	The "maxmean" statistic is used to test the gene set distribution.	No
Gene Set Enrichment Analysis	GSEA	The Kolmogorov-Smirnov statistic is computed to test for uniform distribution of gene sets	No
Gene Set Variation Analysis	GSVA	Quantifying gene set enrichment scores independent of sample labels.	No
Over-representation analysis	ORA	Simple and efficient test based on the hypergeometric distribution.	No
Significance Analysis of Function and Expression	SAFE	Estimating the significance of gene sets by sample permutation.	No
Gene Association Network-based Pathway Analysis	GANPA	Determining gene non-equivalence by the network and assigning gene weights within pathways.	Yes
Gene Graph Enrichment Analysis	GGEA	Estimating the agreement of known regulatory interactions with observed expression data.	Yes
Latent Pathway Identification Analysis	LPIA	Identify GO function and differentially expressed gene weighted pathway network by random walk.	Yes
Network Enrichment Analysis Test	NEAT	According to the number of links of the gene set in the network to verify whether the enrichment of the gene set exists based on the hypergeometric distribution.	Yes
Pathways based on Network information	PathNet	Evaluating hidden pathway dependencies by relying on intra- and inter-pathway connectivity.	Yes
Signaling Pathway Impact Analysis	SPIA	Combining ORA with the probability of propagation of expression changes in pathway topology	Yes

Table S2. Detailed pathway activity assessment results of all methods on BRCA dataset and SARS-CoV-2 dataset.

	M39739	M27888	hsa05224	M41727	M39859	M40066	M42560	M42569	M42580	M42581	hsa05171
Camera	1998	275	2139	966	1925	620	1191	1925	880	1231	24
GSA	1725	312	2047	1712	1736	1054	1146	1240	1757	1576	79
GSEA	1834	562	2181	1539	2076	654	1400	2261	974	1162	22
GSVA	2092	515	2181	863	1904	1196	1637	1631	701	997	28
ORA	2385	627	2385	1618	1502	1759	375	2097	908	2071	762
SAFE	1949	417	1997	1633	1296	1132	957	2047	690	1791	140
GANPA	2171	2000	2208	2097	1786	484	1683	2261	873	1109	346
GGEA	2385	1079	678	1403	1182	718	1169	2261	2261	**684**	256
LPIA	514	1914	501	1601	1210	1356	1471	1160	1949	1450	555
NEAT	200	1288	217	2004	**361**	889	784	**22**	136	767	663
PathNet	861	88	969	428	991	1016	782	2004	1131	1395	30
SPIA	1245	106	1416	2261	2261	**301**	**352**	2261	368	2261	10
ActivePPI (canberra)	300	121	763	860	509	903	1563	70	155	1194	40
ActivePPI (cosine)	246	98	697	237	508	499	1279	71	153	797	44
ActivePPI (euclidean)	**40**	**3**	186	**191**	504	501	567	30	**96**	845	**6**
ActivePPI (manhattan)	143	12	527	191	478	473	1038	31	820	756	9
ActivePPI (minkowski)	42	550	**178**	235	559	556	623	60	138	900	32

Note: The best results for each pathway are bolded.
